# PRAJA is overexpressed in glioblastoma and contributes to neural precursor development

**DOI:** 10.18632/genesandcancer.151

**Published:** 2017-07

**Authors:** Joshua Shin, Viveka Mishra, Eric Glasgow, Sobia Zaidi, Kazufumi Ohshiro, Bhargava Chitti, Amee A. Kapadia, Neha Rana, Lopa Mishra, Chu-Xia Deng, Shuyun Rao, Bibhuti Mishra

**Affiliations:** ^1^ University of Virginia, Charlottesville, VA, USA; ^2^ Massachusetts Institute of Technology, Cambridge, MA, USA; ^3^ Department of Molecular Oncology, Georgetown University, Washington DC, USA; ^4^ Center for Translational Medicine, Department of Surgery, George Washington University, Washington, DC, USA; ^5^ Department of Medicine, George Washington University, Washington, DC, USA; ^6^ John Hopkins University, Department of Chemical and Biomolecular Engineering, Baltimore, MD, USA; ^7^ McLean High School, McLean, VA, USA; ^8^ Faculty of Health Sciences, University of Macau, Macau SAR, China

**Keywords:** PRAJA, glioblastoma, apoptosis

## Abstract

PRAJA, a RING-H2 E3 ligase, is abundantly expressed in brain tissues such as the cerebellum and frontal cortex, amongst others, and more specifically in neural progenitor cells as well as in multiple cancers that include glioblastomas. However, the specific role that Praja plays in neural development and gliomas remains unclear. In this investigation, we performed bioinformatic analyses to examine Praja1 and Praja2 expression across 29 cancer types, and observed raised levels of Praja1 and Praja2 in gliomas with an inverse relationship between Praja1 and apoptotic genes and Praja substrates such as Smad3. We analyzed the role of Praja in the developing brain through loss of function studies, using morpholinos targeting Praja1 in embryonic zebrafish, and observed that Praja1 is expressed prominently in regions enriched with neural precursor cell subtypes. Antisense Praja morpholinos resulted in multiple embryonic defects including delayed neural development likely through increased apoptosis. Further studies revealed high levels of Cdk1 with loss of Praja1 in TGF-β or insulin treated cells, supporting the link between Praja1 and cell cycle regulation. In summary, these studies underscore Praja's role in mammalian brain development and Praja1 deregulation may lead to gliomas possibly through the regulation of cell cycle and/or apoptosis.

## INTRODUCTION

Glioblastoma multiforme (GBM) is the most common primary malignancy of the brain as it is highly infiltrative and has a poor prognosis among cancers. GBM constitutes 17% of all brain cancers, primary and metastatic, with median survival of 14.6 months and a two-year survival rate of 30% [1]. With current therapeutics, improvement in patient survival is marginal, therefore identification of new targets remains urgent. E3 ligases have recently been identified as viable targets in cancer [2, 3]. Yet to date, specific populations responsive to therapy have not been delineated for most cancer types including glioblastomas. We identified a RING E3 ligase, PRAJA that is highly expressed in brain tissues including the cerebellum, cerebral cortex, occipital pole, frontal lobe, amongst others [4]. Interestingly, Praja1 (PJA1) has been shown to be linked to human chromosome Xq12, between markers DXS983 and DXS1216, which is an area linked to X-linked mental retardation (MRX) [4]. Additionally, we have previously demonstrated that PJA1 is involved in regulating tumor suppressors in the Transforming Growth Factor-β (TGF-β) signaling pathway [5-7]. In this investigation, to explore a potential role for PRAJA in tumorigenesis, we conducted a genomic analysis of Praja1 (PJA1), Praja2 (PJA2) and Smad3 across 29 tumor types. One of the most striking observations was that the greatest amplitude in expression of PJA1 and PJA2 occurred in glioblastomas [8], and correlated with a concomitant and significant decrease in expression levels of Smad3, which we have identified as a PJA1 substrate [6, 7], and as a key receptor that regulates TGF-β signaling molecules [9, 10].

A precise sequence of genetic, biochemical, physical and environmental factors lead to the development of accurately placed nerve cells in the cortex, with its dendrites, synapses and axons in a network where each neuron functions as a part of the symphony that makes up the brain [11]. Ubiquitination and apoptosis are major mechanisms that are active amongst neuronal cell precursors and neurons to modulate the population and connectivity of the cerebral cortical neurons. For instance, E3 ligases play a role by the degradation of the pro-apoptotic p53 and a few anti-apoptotic factors. Another example is the degradation of Myc protein by the E3 ligase Huwe1, which allows expression of neurogenic markers (Neurogenin, N-Cadherin and MAP2) normally suppressed by Myc [12]. Here we report the expression of PJA1 E3 ligase in embryonic brain development as well as in TGF-β deficient *Sptbn^−/−^* mutant mice. Loss of PJA1 in a zebrafish model leads to delayed neural development, apoptosis, as well as loss of dorsalization. Further kinases analysis revealed the association of PJA1 with cyclin-dependent kinase 1 (Cdk1, cdc2) activation [13, 14], indicating a functional pathway in cell cycle, apoptosis as well as neural malignancies such as gliomas.

## RESULTS

### Bioinformatic analyses revealed increased levels of PJA1 and PJA2 in brain malignancies

**Figure 1:**
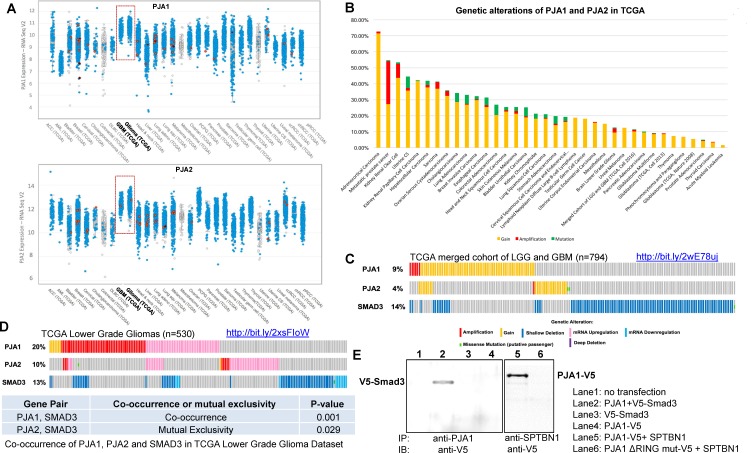
Bioinformatic analyses of PJA1, PJA2 and Smad3 genetic alterations in brain tumors from the databases of The Cancer Genome Atlas **A.** PJA1 and PJA2 RNA expressions shown across 29 cancer types in TCGA databases. RNA expression is shown to be relatively higher in gliomas and glioblastoma multiforme. **B.** Cancer histogram analysis of genetic alterations of PJA1 and PJA2 in 36 cancer types in TCGA databases. **C.** Analyses of PJA1, PJA2, and Smad3 expression in the TCGA merged cohort of LGG and GBM dataset TCGA Brain Lower Grade Glioma (LGG) dataset (*n* = 794). **D.** Analyses of PJA1, PJA2, and Smad3 genetic alterations in the TCGA LGG dataset (*n* = 530). Genetic alterations of PJA1 frequently co-occur with Smad3 alterations (*p* = 0.001) whereas PJA2 alterations are mutually exclusive with Smad3 alterations (*p* = 0.029). **E.** Association of PJA1 with Smad3 and Sptbn1 was examined by co-immunoprecipitation with anti-PJA or anti-SPTBN1 in cells transfected with indicated plasmids and the binding of PJA1 with Smad3 and SPTBN1 was determined by anti-V5.

PJA1 and PJA2 share more than 80% sequence identity of the RING finger domain at the C-terminal. To better understand their roles in brain development and malignancies, we performed bioinformatic analyses of the expression of PJA1 and PJA2 across 29 cancers in the TCGA databases and found that both PJA1 and PJA2 are highly expressed in brain malignancies including Glioblastoma (GBM) and Gliomas (Figure 1A) [8]. The same results were observed in the TCGA adrenocortical carcinoma study and an advanced prostate cancer study [15] (Supplementary Figure 1). We further analyzed genetic alterations of PJA1 and PJA2 that include copy number (CPN) gain, amplification, shallow deletion, deep deletion and mutations in 36 different cancer types. CPN gain and amplification are the most common alterations of these genes across all cancer types analyzed (Figure 1B), suggesting potential oncogenic roles of PJA in cancers. In a TCGA study with merged cohort of Low Grade Glioma (LGG) and GBM [8], PJA1, PJA2 Smad3 and altered in 9%, 4% and 14% of samples, respectively (Figure 1C). Similarly, we observed increased expression of PJA1 and PJA2 in 20% and 10%, respectively, of the 530 samples from 516 sequenced patients in a TCGA study on LGG (Figure 1D). While CPN gain or amplification is frequently observed in PJA1 and PJA2, Smad3 is more frequently downregulated or deleted (Figure 1C-1D). Interestingly, genetic alterations of PJA1 but not PJA2 frequently co-occur with Smad3 alterations, which is consistent with our previous findings that PJA1 directly binds to TGF-β signaling components, Smad3 and its adaptor SPTBN1 (Figure 1E) [7].

### PJA1 is expressed in brain regions enriched with neuronal precursor cells

**Figure 2:**
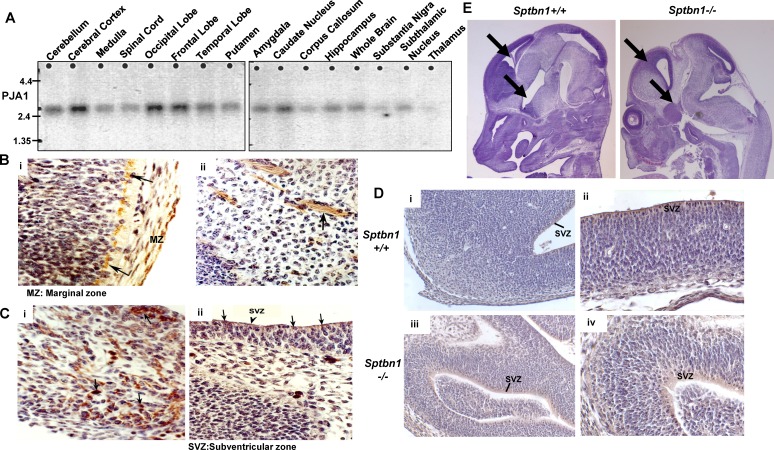
PJA1 expression in brain **A.** Northern blot analysis of PJA1 expression in human brain tissues. Cerebral cortex, occipital, frontal lobes and caudate nucleus demonstrate prominent mRNA for PJA1. **B.** Immunoperoxidase labeling of PJA1 in E12 mouse embryo forebrain marginal zone (MZ) (i) and mantle zone (ii). The left panel (i) shows Cajal-Retzius cells of the marginal layer labeled (arrows) while the right panel (ii) shows fibrillary label extending from cells in the mantle zone. **C.** Immunoperoxidase labeling of PJA1 in E12 mouse embryo midbrain (i) and cerebellum (ii). PJA1 is expressed in the SVZ cells and in developing midbrain nuclei (arrows). **D.** PJA1 expression compared in E12 forebrain of wild type (i-ii) and mutant *Sptbn1^−/−^* mice (iii, iv). Left panels are (i, iii) at 20X magnification; right panels (ii, iv) are at 40X magnification. PJA1 is expressed in SVZ cells and appears to be more widely distributed in embryonic brain of mutant *Sptbn1^−/−^* mice. **E.** Immuno-histochemical analysis of H & E staining of mouse embryos reveals the fore brain development defect in mutant *Sptbn1^−/−^* mice.

Given that PJA1 is highly expressed in brain malignancies, we then examined PJA1 expression in brain tissues. Northern blot analysis of PJA1 mRNA demonstrates a 2.7 kb transcript in human brain tissues and is found to be more abundantly expressed in the cerebral cortex, occipital lobe, frontal lobe, cerebellum and caudate nucleus. Relatively low abundance of the PJA1 transcript is seen in the brainstem structures, such as the medulla, spinal cord, corpus callosum and substantia nigra (Figure 2A). Additional immunohistochemistry (IHC) staining of PJA1 in normal brain revealed that PJA1 is expressed in the hindbrain, midbrain, forebrain, cerebellum, and basal ganglia of E12 and E15 (12 and 15 days post-coitum) embryonic mice (Figure 2B-2C). Earlier, day 11.5 embryos were found to express PJA1, in the Subventricular Zone (SVZ) cells. PJA1 label is observed in cells that are identified as Cajal-Retzius cells by morphology and location [16], in the outer layer (marginal zone) of the E12 developing cortex in mouse embryo brain (Figure 2B, i). PJA1 label is also demonstrated in the cells of the SVZ in E12 forebrain cortex (Figure 2C ii). In both cell types, the label appears perinuclear and polarized. In the developing forebrain cortex, PJA1 label also appears in fibrillar form extending from cells with larger nuclei (Figure 2B ii). This fibrillary appearance is also observed with the TGF-β adaptor, SPTBN1 labels at the same embryonic stage of mouse brain development. In the midbrain, cerebellum, and hindbrain, PJA1 is expressed in the cytoplasm of scattered cells among the developing midbrain and brainstem nuclear groups (Figure 2C i). However, SVZ cells continue to express PJA1 (Figure 2C ii).

Consistently, PJA1 IHC in different areas of E12 brain demonstrates that PJA1 is normally expressed in the SVZ that is enriched with neuronal precursor cells [17] and the collection of hindbrain nuclei in E12 embryonic brain of wild type mouse embryo (Figure 1D i, ii). Interestingly, in contrast to that of wild type mice, PJA1 expression is more widely expressed in the developing brain of TGF-β deficient *(Sptbn1^−/−^)* mutant mice. Additionally, PJA1 expression appears to be strongly diffused in *Sptbn1^−/−^* mutant mice compared with that in wild type SVZ cells (Figure 1D iii, iv). It is noteworthy that the mutant embryo brain exhibits multiple fore brain developmental defect including abnormal brain vesicles, further suggesting an important role of PJA1 in normal brain development (Figure 1E) [10, 18].

### Loss of PJA1 leads to developmental defect in zebrafish embryonic brain with increased apoptosis

**Figure 3:**
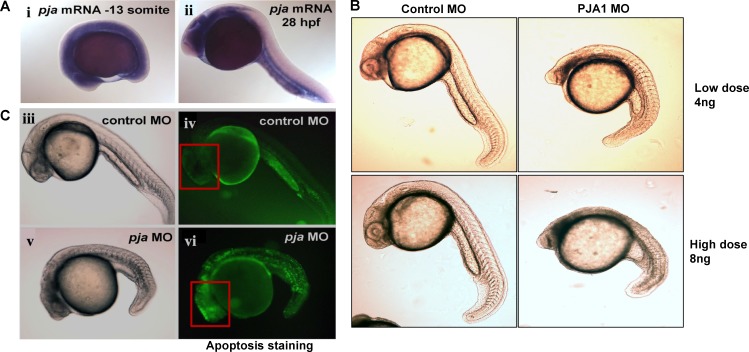
Apoptosis dramatically increases in zebrafish embryos upon PJA1 knockdown **A.** PJA1 mRNA expression was determined by whole-mount *in situ* hybridization in developing embryos. PJA1 mRNA is expressed in the yolk and ventral side of the embryo at the 13 somite stage (i) and in intestinal and vascular tissues at 28 hour post-fertilization (hpf) (ii). **B.** Developmental defects were observed in zebrafish embryos injected with 4ng or 8ng of antisense PJA1 morpholino oligonucleotides (MO) but not in embryos injected with same dose of control MO. **C.** Apoptosis was visualized by acridine orange staining in live embryos, where apoptotic cells are indicated by green fluorescence. Compared to that of the control MO injected embryos at 24 hpf. **D.** injection of PJA1 MOs severely disrupted embryogenesis (v) and resulted in high levels of apoptosis (vi). Red squares indicate the embryonic brain of zebrafish.

We also detected normal PJA1 expression in zebrafish embryo (Figure 3A) using whole mount *in situ* hybridization. PJA1 is expressed in the yolk sac and is evenly and strongly expressed along the entire length of the embryo, with a steep ventral-dorsal gradient such that high ventral expression abruptly drops to undetectable levels in the dorsal-most 20-30% of the embryo. By 28 hours post-fertilization (hpf), the PJA1 expression pattern becomes more refined and restricted to specific tissues. In the brain, PJA1 is strongly expressed throughout the basal plate, while expression in the alar plate is predominantly restricted to the ventricular zones. In addition, PJA1 is expressed in the head mesenchyme, intestinal and vascular structures of the trunk and tail, and mesendodermal tissues near the yolk sac (Figure 3A), indicating that PJA1 may play a particularly important role in GI organ development.

To test our hypothesis that PJA1 is required for normal brain development, translation-blocking antisense PJA1 Morpholino oligonucleotides (MO) were injected into single-cell stage zebrafish embryos to knock down PJA1 that is normally expressed in zebrafish embryos (Figure 3A). As we expected, morpholino-mediated PJA1 knockdown results in multiple developmental defects throughout the embryo (Figure 3B) including reduced brain development, shortened and deformed tail, abnormal notochord, and loss of the chevron shape of the somites. The overall appearance of the PJA1 MO injected embryos (*Praja1* morphants), in comparison to control MO injected embryos, is that they have smaller head size with curved bodies and tails (Figure 3B-3C). Further, we found increased apoptosis visualized by acridine orange staining throughout the somitogenesis period in PJA1 MO injected embryos relative to control MO injected embryos. By 24 hpf, when somitogenesis is complete and most organ primordia have formed, apoptosis remains high, specifically in the head (Figure 3C iv, vi); by 36 hpf, apoptosis in PJA1 morphants starts to level off; and by 48 hpf is only slightly higher than that in control embryos. These studies indicate that PJA1 is important for inhibiting apoptosis during zebrafish embryogenesis.

### PJA1 protects cells from apoptosis

**Figure 4:**
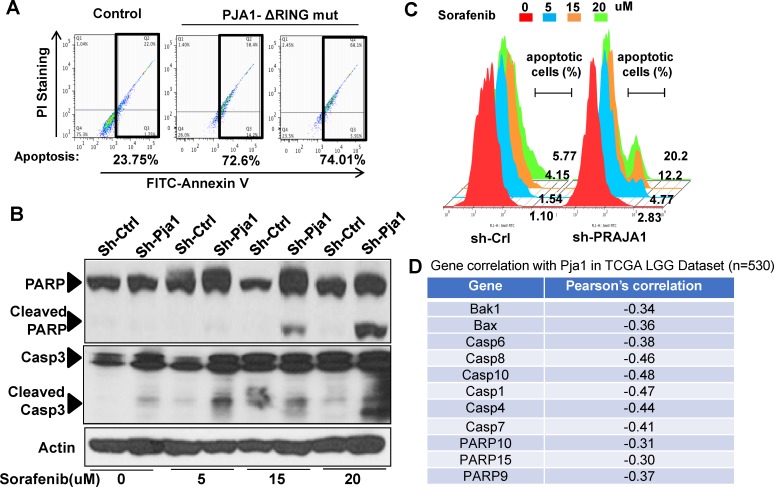
PJA1 defects lead to increased cell apoptosis **A.** Apoptotic response to Cisplatin of the Huh7 cells that express a RING domain-deleted PJA1 mutant. The expression of the inactive PJA1 mutant (ΔRING mut) causes more than a three-fold increase in the percentage of apoptotic cells. **B.** Apoptotic response of HepG2 cells with reduced PJA1 expression after treatment with Sorafenib. PJA1 knockdown demonstrated that Caspase3 and PARP cleavage were significantly elevated compared to control with a dose-dependent pattern. **C.** The percentage of apoptotic cells is consequently higher in PJA1 knockdown and Sorafenib treatment. **D.** Negative Pearson's correlation between PJA1 and apoptotic genes in TCGA LGG dataset.

To further examine the potential mechanism for the developmental defects caused by PJA1 inhibition and determine the role of PJA1 in cellular apoptosis, we introduced RING domain-deleted mutant PJA1 (ΔRING mut) in cells. This mutant was identified as a loss-of-function mutant by our group in the regulation of TGF-β signaling through Smad3 and its adaptor, SPTBN1 [7, 19, 20]. Through Annexin V staining, we found that expression of PJA1 mutant in cells results in three-fold increase of apoptosis (Figure 4A). In addition, knockdown of PJA1 by shRNA in HepG2 cells leads to increased cell apoptosis as determined by increased PARP expression as well as increased Caspase 3 and cleavage of Caspase3 (Figure 4B). To examine whether kinases are required for apoptosis induced by PJA1 defect, we treated cell with Sorafenib, a tyrosine kinase inhibitor. Interestingly, Sorafenib synergizes with PJA1 knockdown to increase cell apoptosis in a dose-dependent manner (Figure 4B-4C). Consistently, analysis of co-expression of genes with PJA1 revealed a negative Pearson's correlation between PJA1 and several apoptotic genes, including Bax and Bak as well as caspases 6, 8, 10, 1, 4 and 7 in LGG (Figure 4D). All these data strongly support the anti-apoptotic effect of PJA1.

### PJA1 is associated with CDK1 activation

**Figure 5:**
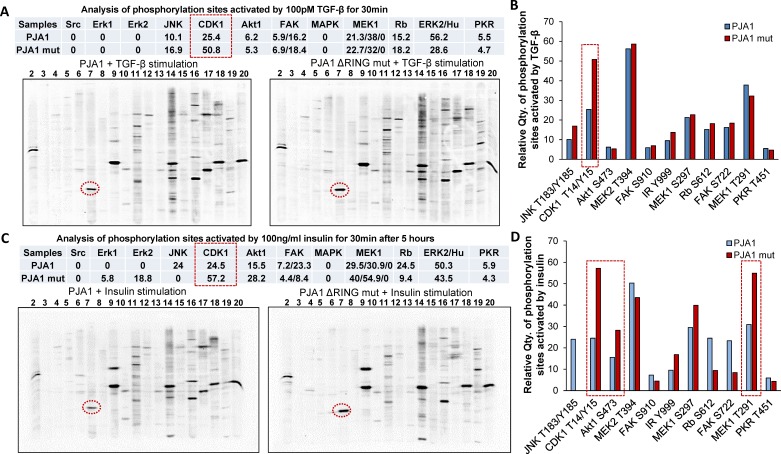
Elevated Cdk1 activity is associated with loss of PJA1 **A.** Increased Cdk1 phosphorylation in cells transfected with PJA mutant. Protein phosphorylation visualized by ECL show increased phosphorylation activity in Cdk1 (Lane 7) in TGF-β treated cells transfected with PJA1 mutant (ΔRING mut) (right) compared to that in cells transfected with wild type PJA1 setting (left). The quantification results of bands were showed in the top table. **B.** Bar graph shows the quantification of the phosphorylation of each kinase and their respective sites as indicated in A. **C.** Increased phosphorylation activity in Cdk1 (Lane 7) in insulin treated cells transfected with PJA1 mutant (ΔRING mut) (right) compared to cells transfected with wild type PJA1 setting (left). The quantification results of bands were shown in the top table. **D.** Bar graph shows the quantification of the phosphorylation of each kinase and their respective sites as indicated in **C**.

To further explore the mechanism underlying PJA1-mediated brain development, we performed Kinetworks^TM^ phosphor-site broad coverage pathway screen in cells transfected with either wild type PJA1 or PJA1 ΔRING mut, a loss-of-function mutant [7]. Profiles of activated kinases, induced by either TGF-β or insulin treatment, were examined through band visualization *via* ECL and quantification (Figure 5A-5D). We observed consistently a two-fold induction of Cdk1 phosphorylation at T14/Y15 in both TGF-β and insulin treated cells transfected with PJA mut compared with cells transfected with PJA wild type (Figure 5, Supplementary Tables 1-4), indicating that Cdk1 activation is negatively regulated by PJA1. Our study is in line with previous findings showed that aberrant Cdk1 activation has been associated with neuronal cell apoptosis and Cdk1 is critical to brain development [13, 14, 21-23]. In addition to Cdk1 activation, insulin activating through insulin receptors induces activation of MEK/ERK and Akt signaling pathway in cells expressing PJA1 mutant, suggesting a negative regulation of these pathways by PJA1 as well.

## DISCUSSION

In this study, we explored the role of praja1 (PJA1) in brain development and found that PJA1 protects cells from apoptosis and is required for the normal embryonic development. In addition, loss of PJA1 leads to hyper-activation of Cdk1 and thereby induces cell apoptosis during early embryogenesis possibly through cell cycle mitosis stress as Cdk1 is known to interact with cylin B1 to control the progression of cell cycle from G2- to M-phase. Aberrant Cdk1 activation has been associated with cell apoptosis [13, 14, 21-24].

Our integrated genomic analyses of LGG and GBM indicate a potential new role for PJA1 in gliomas, potentially through its preciously demonstrated ubiquitination of the Smad3-likely reflecting the TGF-β tumor suppressor role in this cancer [5-7, 9, 10, 18]. Interestingly, although we observed PJA2 mutations in brain cancer [8], we found no mutations in PJA1, only alterations leading to raised PJA1 expression. Further insight into PJA1 was obtained by examining its co-expression with apoptotic genes. Pearson's correlation analysis revealed a negative moderate relationship between PJA1 and these apoptotic genes, which suggest an negative anti-apoptotic role of PJA1. Indeed, PJA1 knockdown leads to high levels of apoptosis. This pro-survival, anti-apoptotic role for PJA1 lends further support to the notion that it may be function as an oncogene.

The study suggests important functions for PJA1 in the neural development as well. The appearance of PJA1 label in the region enriched with neural progenitors (SVZ cells) without labeling radial glial cells and other progenitor cells, characterizes a subset of progenitor cells by phenotype. Cajal Retzius cells and subventricular zone cells are predominant early neural progenitor cells of embryonic brain. Usually also confirmed by double label with ‘Reelin’ [25]- Cajal Retzius cells are located in the outer zone of the developing cortex, and have been shown to differentiate into neuronal cell phenotype [26]. SVZ cells on the other hand are nestin [27] positive, *musashi-*1 [28] positive, and give rise to radial glial cells that in turn populate the embryonic forebrain cortex with neuroblasts.

Loss of PJA1 expression in zebrafish led to apoptosis, resulting in delayed neural development. The upregulation of Cdk1 in loss of PJA1 function suggests a key role of loss of PJA1 expression in the irregularities of Cdk1/cyclin B mitotic cascade that instigates hyper-phosphorylation of neuronal proteins and neuronal apoptosis. Interestingly, previous research has marked that Cdk1/cyclin B overexpression in gliomas, marking it as a potential oncogene and highlighting the various apoptotic roles that Cdk1/cyclin B plays contingent upon Praja expression [13, 14].

PJA1's role in neural development and gliogenesis can be understood through its structural and functional homolog PJA2 and its role as an A-kinase-anchoring protein (AKAP), which anchors protein kinase A1 (PKA) to specific intracellular sites and is involved in protein sorting at axosomatic synapses [29]. PKA and cAMP also serve roles in axon formation as impeding PKA anchored to AKAPs increased cAMP gradient in neurons which led to enhanced axon elongation [30]. PJA1 appears to play a multifunctional role in neuronal development. The MAGE (melanoma antigen) member NRAGE (neurotrophin receptor associated MAGE famohomologue), also known as Dlxin-1 in mouse and MAGE-D1 in human [31], is directly targeted by PJA1 for ubiquitination and subsequent degradation [32]. NRAGE deletion impedes BMP regulated neural developmental apoptosis of sympathetic neurons [33]. In addition, NRAGE has also been found to inhibits several AKAPs in conjunction with p75 neurotrophin receptor (p75NTR) [34]. A recent study found that PJA2 induces ubiquitin/proteasomal degradation of Mob1 (Mps one binder 1), a kinase regulator of the Hippo cascade, promoting glioblastoma formation [35]. Collectively, our studies demonstrate an important role for Praja in neural development, and gliomas, providing a basis for future investigations into targeting Praja in gliomas, that are lethal cancers.

## MATERIALS AND METHODS

### Bioinformatics analysis

The Cancer Genome Atlas (TCGA) database from cBioportal was utilized to examine transcriptomic and genomic alterations of Praja1 (PJA1), Praja2 (PJA2), and Smad3 (SMAD3) (http://www.cbioportal.org/). Genomic profiles were chosen based on our studies of interest including TCGA Brain Lower Grade Glioma (LGG), consisting of 530 samples from 516 patients, and TCGA Merged cohort of LGG and Glioblastoma Multiforme (GBM), consisting of 794 samples. Mutations, putative copy number alterations from GISTIC, mRNA expression z-scores (RNA Seq V2 RSem; z-score threshold ± 2), and protein expression z-scores (z-score threshold ± 2) were chosen out of the profiles for TCGA Lower Grade Glioma and TCGA merged cohort of LGG and GBM. PJA1 and PJA2 expression were first analyzed across all cancer types. Oncoquery language was utilized for the selection of samples with gain of function (GAIN), amplification (AMP), mRNA upregulation (EXP > = 1.5), or mutations in PJA1 or PJA2 (MUT), as well as deletion or loss (HETLOSS, HOMEDEL), mRNA downregulation (EXP < = −1.5), or mutation in Smad3 when observing TCGA Lower Grade Glioma. Following the completion and submission of the outlined query, the oncoprint, mutations, mutual exclusivities, and mRNA coexpression tabs were chosen and their results were individually recorded. Specifically, when examining the oncoprint results, the percentage of patient samples with alterations in the queried genes and whether those various samples increased expression of the genes (i.e. amplification, gain, or mRNA upregulation) or decreased expression (i.e. deletion, loss, or mRNA downregulation) was noted and observed. Subsequently, we attempted to identify mutations in PJA1 and PJA2 in these malignancies, thereby juxtaposing the results and structures of the two genes. While examining the mutual exclusivity or co-expression of PJA1, PJA2, and Smad3 in the TCGA Lower Grade Glioma dataset, the statistical significance of these relationships was noted. Additionally, while utilizing mRNA co-expression from the TCGA Lower Grade Glioma dataset, Pearson's correlation between PJA1 and genes (Caspases 1, 4, 7, 6, 8, 10) implicated in apoptosis were noted.

### Northern blot analysis

Northern blots with 2 *μ*g poly-A(+) mRNA from human brain (Clontech, USA) were probed with ^32^P-labeled Praja Clone CH7 [5] insert antisense strand using ExpressHyb hybridization solution (Clontech, USA) at 68°C. They were washed according to the manufacturer's instructions and subjected to autoradiography. A ^32^P-labeled β-actin probe supplied with the Northern blots was used as a control to confirm that normalized RNA levels were present in each lane (Clontech).

### Embryonic tissue preparation

129SvEv/Black Swiss Mice were used following an institutionally approved animal protocol in accordance with the requirements and recommendations of the NIH “*Guide for the Care and Use of Laboratory Animals*.” The age of the embryos was determined by post-appearance of the vaginal plug (day 0). Noon of this day was considered 0.5 days of gestation post-coitus (p.c). The embryos were dissected at transitional times between days of 9.5 and 14.5 p.c. The identification and isolation of embryos were carried out under an operative microscope. The specimens were fixed in FEA fixative (4% formaldehyde, 85% ethanol and 5% glacial acetic acid) and embedded in paraffin [36]. The sections, 8-15 μM thick, were cut in the longitudinal or transverse planes using a microtome (Reichert-Jung, USA).

### Immunohistochemical staining

An indirect immunoperoxidase procedure was used for immunohistochemical localization of Sptbn1 and PJA1 protein in brain tissues of mouse embryos. Antibody to a peptide corresponding to amino acids 145-149 (CLRRKYRSREQPQS) of PJA1 was produced in rabbit, and used to detect Praja by immunohistochemical labeling [5]. For β2SP, the primary antibody used was rabbit antimouse EL-1 (against N-terminal aminoacids 2-14, ELQRTSSVSGPLS) as described previously [7, 19, 20]. Sections (8 μM) were treated with xylene to remove paraffin, and the tissue was dehydrated by placing it stepwise into graded higher concentrated alcohol and rinsed in 1x phosphate-buffered saline (PBS). Endogenous peroxidase was quenched using 3% hydrogen peroxide (Sigma). Non-specific binding sites were blocked with 1x PBS containing 5% goat serum and 1 mg/ml bovine serum albumin (BSA). The sections were incubated overnight at 4°C in humidor with primary antibody diluted to 2.5-5 μg/ml in 1x PBS containing 1 mg/ml BSA and 0.05% Triton X-100. All subsequent steps were done at room temperature, including four 5-min rinses with 1x PBS containing 1% goat serum after each successive step. Sections were incubated with peroxidase-conjugated goat anti-rabbit antibody (Jackson Immunoresearch Laboratories, West Grove, PA) that was diluted in 0.05M PBS in 1% serum for 30 min. After rinses, 200-500 μl of the insoluble peroxidase substrate DAB (Sigma Fast) was added to cover the entire tissue on the slide. Color development was monitored under the microscope, and then slides were rinsed for 2 min in distilled water. Counterstaining was then performed with Harris's hematoxylin solution (Sigma) (modified) for 1 min, followed by a rinse in distilled water for 5 min. Sections were dehydrated by passage through graded alcohol concentrations and xylene. The coverslips were mounted using DPX (Fluka Labs).

### Morpholino injection in zebrafish embryos

Morpholinos were synthesized and purchased from Gene Tools (http://www.gene-tools.com/). A stock solution of 10μg/μl was prepared by dissolving the lyophilized powder in twice-distilled water. The stock solution was diluted to a working concentration of 2μg/μl in 1 × Danieau's solution. Freshly laid zebrafish embryos were collected and placed into embryo culture medium and transferred onto an agarose platform. 4ng or 8ng of morpholinos targeting PJA1 or control mopholinos was injected in the yolk of embryos. Microinjection was performed using a Nanoject injector II.

### Praja function and loss of function proteomics

Cultured cells transfected with wild type PJA1 or PJA1 ΔRING mut were treated with either TGF-β (100pm) for 30min or insulin (100ng/ml) for 30min after 5h starvation. Then cells were lysed with RIPA lysis buffer supplemented with phosphatase and protease inhibitors. Protein concentrations were determined by Bio-Rad Bradford assay prior Kinetworks^TM^ phosphor-site broad coverage pathway screen (Kinexus Bioinformatics Corporation). Protein phosphorylation activities were analyzed, including the combination of both analytical techniques such as immunoblotting, protein visualization through enhanced chemiluminescence (ECL), and gel electrophoresis, as well as assorted methodologies. Thus, the target kinases including Src, Erk1, Erk2, Jun, Cdc2, Akt1, FAK, MAPK, MEK1, Rb, ERK2/Hu, and PKR were determined by the KCPS-1.0 screen. Through the process, an adequate reading of the quantities was observed over a 2000-fold linear range.

## SUPPLEMENTARY MATERIALS FIGURE AND TABLES



## Abbreviations

GBM: Glioblastoma multiforme
MRX: moiX-linked mental retardation
TGF-β: Transforming Growth Factor-β
PKA: protein kinase A1
AKAP: A-kinase-anchoring proteins
Cdc2: cell division cycle protein 2 homolog
Cdk1: cyclin-dependent kinase 1
LGG: Lower Grade Glioma
TCGA: The Cancer Genome Atlas
SVZ: Subventricular Zone
MO: Morpholino oligonucleotides
ISHH: *in situ* hybridization histochemistry
hpf: hours post-fertilization
PBS: Phosphate buffered saline
ECL: enhanced chemiluminescence
RIPA: radioimmunoprecipitation
BSA: bovine serum albumin
MAGE: melanoma antigen
NRAGE: neurotrophin receptor
