# PRAJA is overexpressed in glioblastoma and contributes to neural precursor development

**DOI:** 10.18632/genesandcancer.158

**Published:** 2017-09

**Authors:** Joshua Shin, Viveka Mishra, Eric Glasgow, Sobia Zaidi, Kazufumi Ohshiro, Bhargava Chitti, Jian Chen, Amee A. Kapadia, Neha Rana, Lopa Mishra, Chu-Xia Deng, Shuyun Rao, Bibhuti Mishra

**Affiliations:** ^1^ University of Virginia, Charlottesville, VA, USA; ^2^ Massachusetts Institute of Technology, Cambridge, MA, USA; ^3^ Department of Molecular Oncology, Georgetown University, Washington, DC, USA; ^4^ Center for Translational Medicine, Department of Surgery, George Washington University, Washington, DC, USA; ^5^ Department of Medicine, George Washington University, Washington, DC, USA; ^6^ John Hopkins University, Department of Chemical and Biomolecular Engineering, Baltimore, MD, USA; ^7^ McLean High School, McLean, VA, USA; ^8^ Faculty of Health Sciences, University of Macau, Macau SAR, China; ^9^ Department of Gastroenterology, Hepatology, & Nutrition, The University of Texas M. D. Anderson Cancer Center, Houston, Texas, USA

**This article has been corrected:** Dr. Jian Chen was added to the author list.

The authors sincerely apologize for this oversight.

Original article: Genes&Cancer. 2017;8:640-649. https://doi.org/10.18632/genesandcancer.151.

PMID: 28966725; PMCID: PMC5620009

